# Understanding Physical Functioning in Patients with Sarcoma After Amputation: Using an International Classification of Functioning, Disability and Health (ICF) Approach

**DOI:** 10.1245/s10434-026-19373-y

**Published:** 2026-04-05

**Authors:** Tom I. Bootsma, Dide den Hollander, Milou J. P. Reuvers, Ingrid M. E. Desar, Roger Wilson, Michiel A. J. van de Sande, Winan J. van Houdt, Ingrid C. M. van der Geest, Bart H. W. B. Schreuder, Suzanne E. J. Kaal, Johannes J. Bonenkamp, Winette T. A. van der Graaf, Olga Husson

**Affiliations:** 1 Department of Public Health, Erasmus University Medical Center, Rotterdam, The Netherlands; 2 Department of Medical Oncology, Netherlands Cancer Institute, Antoni van Leeuwenhoek, Amsterdam, The Netherlands; 3 Department of Medical Oncology, Radboud University Medical Center, Nijmegen, The Netherlands; 4 Sarcoma Patient Advocacy Global Network, Wölfersheim, Germany; 5 Department of Orthopaedic Surgery, Leiden University Medical Center, Leiden, The Netherlands; 6 Department of Surgical Oncology, Netherlands Cancer Institute, Antoni van Leeuwenhoek, Amsterdam, The Netherlands; 7 Department of Orthopaedic Surgery, Radboud University Medical Center, Nijmegen, The Netherlands; 8 Department of Surgery, Radboud University Nijmegen Medical Center, Nijmegen, The Netherlands; 9 Department of Medical Oncology, Erasmus University Medical Center, Rotterdam, The Netherlands; 10 Department of Surgical Oncology, Erasmus University Medical Center, Rotterdam, The Netherlands

**Keywords:** Extremity sarcoma, Amputation, Health-related quality of life, Physical functioning, Patient-reported outcomes, PRO development, EORTC QLQ-C30, TESS

## Abstract

**Background:**

Physical functioning (PF) is significantly affected following amputation for extremity/pelvic sarcomas. Commonly used health-related quality of life (HRQoL) measures such as the European Organisation for Research and Treatment of Cancer Quality of Life Questionnaire (EORTC QLQ-C30) (C30) and the Toronto Extremity Salvage Score (TESS) have not been validated in this population. This study examines the challenges of measuring PF in patients with extremity/pelvic sarcoma after amputation. It explores how these patients interpret the TESS and C30, particularly regarding prosthesis and assistive device use. PF is considered within the International Classification of Functioning, Disability and Health (ICF) framework.

**Patients and Methods:**

We conducted semistructured interviews with 20 patients who had undergone amputation for extremity/pelvic sarcoma, recruited from three Dutch sarcoma centers. Interviews explored patients’ experiences of PF using the C30 and TESS. Thematic analysis was conducted, with data organized into key themes and subthemes within the ICF framework.

**Results:**

Amputation impacts five interrelated ICF domains: body functions and structures, activity and participation, and environmental factors. Within body functions and structures, patients reported challenges related to sensory functions and pain, movement functions, and genital and reproductive health. In the domain of activity and participation, limitations were noted in general tasks and demands, mobility, self-care, domestic life, and community, social, and civic life. Environmental factors such as products and technology and social support also played a role.

**Conclusions:**

A tailored measurement strategy is needed for patients with sarcoma post-amputation. Importantly, neither the C30 nor the TESS explicitly accounts for these contextual factors, which can lead to inconsistent or inaccurate scoring of physical functioning in this population.

**Supplementary Information:**

The online version contains supplementary material available at 10.1245/s10434-026-19373-y.

Sarcomas are rare mesenchymal tumors of soft tissue or bone that can occur at any anatomical location. In the case of soft tissue sarcoma (STS), there is a predilection for the lower (30%) and upper (12%) limbs,^1–4^ while in case of bone sarcoma (BS), the lower (47%) and upper (23%) extremities are more often affected, with occasional involvement of the pelvic bones.^3–6^ Limb-sparing surgery (LSS) has replaced amputation as the cornerstone of treatment.^7–9^ Advances in imaging for staging, surgical reconstructions, and multimodal treatments have led to comparable long-term survival, provided that appropriate surgical margins are achieved and postoperative limb function is maximized.^7–9^ In STS, LSS is combined with (neo-)adjuvant radiotherapy,^10,11^ or in specific cases, with (neo-)adjuvant chemotherapy^12^ or isolated limb perfusion (ILP).^13^ In BS, surgery only is standard treatment in the majority of chondrosarcomas as no systemic treatments are effective, while in osteosarcoma and Ewing sarcoma, the highest survival rates are achieved when combining LSS with neoadjuvant and adjuvant combination chemotherapy regimens.^14^ However, primary or secondary amputation is warranted in a minority of cases, i.e., when LSS is not possible due extensive tumor invasion, expected loss of limb, or failure of prior reconstruction. Additional indications include local recurrence, endoprosthetic failure, intractable wound complications, inadequate soft tissue coverage or palliation in cases of severe pain, bleeding, fungating tumors, or pathologic fractures.^8,15–20^

Patient-reported outcomes (PROs) are frequently assessed among patients undergoing amputation for extremity/pelvic sarcoma. Most often, health-related quality of life (HRQoL), representing the patient’s general perception of the effect of illness and treatment on physical, psychological, and social aspects of life, is assessed.^21^ The physical functioning HRQoL domain includes the ability to perform activities of daily living.^22^ Studies using PROs in extremity and pelvic sarcoma patients focus on the comparison of outcomes between patients who had undergone LSS versus amputation. In patients with extremity STS, no significant differences were observed across most HRQoL domains. However, physical functioning (PF) was generally superior in those who underwent LSS, as reported in the majority of studies.^23^ In patients with osteosarcoma, similar HRQoL outcomes between the two treatment groups were found.^24^ Several studies found that PF is impacted by amputation level: more proximal levels were significantly associated with lower PF scores.^25–27^ A systemic review of HRQoL in patients with extremity BS showed large variety in scores. This review reported that patients who underwent LSS had slightly higher scores on mental dimensions and the mean Mental Component Summary (MCS) score than on the physical dimensions and the mean Physical Component Summary (PCS) score of the Short Form (36) Health Survey (SF-36), compared with patients who underwent amputation.^28^ Kask et al. investigated how functional outcomes were measured in studies among patients with surgically treated lower-extremity STS. The most frequently used patient-reported outcome measure (PROM) was the Toronto Extremity Salvage Score (TESS), but few studies included amputee patients.^29^ More recently, a study comparing PROs in patients with advanced extremity STS who underwent ILP and resection or amputation found very low scores on the physical and role functioning scale of the European Organisation for Research and Treatment of Cancer Quality of Life Questionnaire (EORTC QLQ-C30) (C30) in the amputee group, whereas the other scales of this measure were not affected.^30^ The TESS and C30 are not formally validated in patients with sarcoma who have undergone limb amputation. This raises the question of how amputee patients interpret and complete existing questionnaires (e.g., function with or without prosthesis).

This study examines the challenges of measuring PF in patients with extremity or pelvic sarcoma who have undergone amputation. It explores how these patients interpret the TESS and C30 questionnaires, particularly regarding prosthesis and assistive device use. PF is considered within the International Classification of Functioning, Disability and Health (ICF) framework developed by World Health Organization, which provides a standardized, holistic approach to describing health and functioning.^31^

## Patients and Methods

### Study Design and Participants

This analysis is part of a larger mixed methods study to determine a HRQoL measurement strategy in adult patients with sarcoma (clinicaltrials.gov NCT04071704).^32^ This EORTC VOICE study was amended to include evaluation of HRQoL and its measurement in Dutch patients with STS and BS who had undergone amputation.

Inclusion criteria were: (1) confirmed diagnosis of extremity or pelvic sarcoma (according to the ICD-10 codes C40 and C41.4 for BS, and C49.1 and C49.2 for extremity STS); (2) having undergone amputation for extremity or pelvic sarcoma (either primary or delayed); (3) age ≥ 18 years at diagnosis, because the C30 was only validated among patients with cancer aged 18 years and older; and (4) having the cognitive capacity to provide informed consent and participate in the study (as determined by their treating physician). Exclusion criteria were: (1) being too ill (on the judgment of the treating physician) and (2) the following diagnoses: Kaposi sarcoma and benign and locally aggressive mesenchymal tumors. Purposive sampling was used to ensure that different amputation levels were included, i.e. toes, fingers, partial hand or foot, below knee or elbow (including hand or foot), through knee or elbow, above knee or elbow, disarticulation, and hindquarter or forequarter amputation. Only patients treated within one of the following sarcoma expertise centers (The Netherlands Cancer Institute [Amsterdam], Radboud University Medical Center [Nijmegen], and Leiden University Medical Center [Leiden]) were recruited. Eligible patients received an invitation letter from their (ex-)treating physician explaining the goals and procedure of the study. All participants gave written informed consent prior to the interview. Sociodemographic data were obtained from the participants at the start of the interview. Clinical data were collected from the medical file by a member of the study team.

### Interviews

Semistructured interviews were conducted by trained qualitative researchers (TB, MR) between May and August 2021, by telephone or Microsoft Teams because of physical distancing recommendations due to the coronavirus disease 2019 (COVID-19) pandemic.^33^ The duration of the interviews was on average 75 min and ranged from 45 min to 107 min. Participants were asked an open-ended question regarding their cancer history and experience; use of prosthesis, assistive devices, or other support; and then prompted according to an interview schedule (S1).

### Questionnaires

During the interview, the participant was asked to review the C30 and TESS. The C30 version 3.0 was developed to measure HRQoL in patients with cancer.^34^ This 30-item questionnaire consists of five functional scales (physical, role, cognitive, emotional, and social), a global quality of life scale, three symptom scales (fatigue, pain, nausea and vomiting), and single items assessing common symptoms (dyspnea, loss of appetite, sleep disturbance, constipation, and diarrhea) and perceived financial impact of the disease. The TESS is a physical function measure developed specifically for patients undergoing surgery for extremity tumors.^35^ Different versions are available for upper (29 items) and lower (30 items) extremities.

All items of C30 and TESS were scored during the interviews on a 4-point scale: (1) not at all relevant; (2) a little relevant; (3) quite relevant; and (4) very relevant. Relevance refers to the frequency with which a specific item occurs, and when it occurs, the trouble it may cause. This means the more frequently an item occurs and the more trouble it causes, the more relevant it will be. Finally, the participants were asked whether items could be deleted or other HRQoL issues that they believe to be important should be added to the questionnaires, and what questionnaire they would recommend to measure physical functioning.

### Data Analysis and Reporting

Interviews were recorded and transcribed verbatim anonymously. The transcripts were analyzed by two independent coders (DdH and MR) in NVIVO 14 during data collection, using thematic analysis of Braun and Clark.^36^ Data related to the study aim were grouped in key themes by two coders (DdH and TB) within the framework of physical functioning of the ICF.^31^ Coders discussed and resolved differences, and redefined themes until consensus was reached. To enhance rigor, the coding process included regular consultations with the senior author (OH) to resolve uncertainties and validate interpretative decisions. A patient expert (RW) was engaged to provide feedback on the relevance and clarity of emerging themes, strengthening the patient-centered validity of the analysis. We applied a stratification matrix to ensure diversity in amputation levels. Thematic saturation was reached when no new codes emerged after two or more interviews per level. Recruitment for minor upper extremity and pelvic amputations stopped earlier due to rarity and fewer issues identified. No cases were included for through-elbow upper extremity amputations or lower extremity disarticulation. The original quotes were collected in Dutch. To ensure accurate and consistent translations, an artificial intelligence (AI)-based translation tool was used to translate the anonymized quotes into English. To maintain the integrity and cultural sensitivity of the content, the translations were reviewed by a native-English-speaking co-author (RW).

### Ethics

Ethical approval was obtained from the Institutional Review Board of the Netherlands Cancer Institute (IRB18121), as the institution’s medical ethics committee determined that the study falls outside the scope of the Dutch Medical Research Involving Human Subjects Act (WMO). Additionally, the study was reviewed and approved by national regulatory authorities and local medical ethics committees in each participating institution, in accordance with local regulations.

## Results

### Participants

A total of 20 patients who had undergone amputation for sarcoma (7 upper extremity [3 BS/4 STS], 12 lower extremity [8 BS/4 STS], and one pelvic [BS]) were interviewed; 11 patients used a prosthesis, 7 did not, and in 2 patients this was not applicable as they had undergone a minor amputation (partial hand or foot). In total, 12 patients were male, and the ages of all patients ranged from 25 years to 83 years. More clinical and sociodemographic characteristics are listed in Table 1.

**Table 1:** Patient characteristics

		Upper extremity (*N* = 7)	Lower extremity (*N* = 12) + pelvic bones (*N* = 1)
*Sociodemographic characteristics*	*n*		*n*
Gender	Male	4	8
	Female	3	5
Age at time of diagnosis (years)	Median (range)	48 (22–66)	48 (26–79)
Marital status	Single	1	1
	Married/living with partner	5	12
	Partner not living together	1	0
Caring responsibilities children under 18 years	Yes	2	3
Highest education	High school	2	0
	College or university	5	13
Employment^a^	Full time	1	3
	Part time	3	3
	Unemployed	0	1
	Retired	1	3
	(Partial) Disabled	3	4
	Sick leave	1	0
*Clinical characteristics*		*n*	*n*
Sarcoma histology BS	Chondrosarcoma	1	5
	Osteosarcoma	2	3
	Sarcoma NOS	0	1
Sarcoma histology STS	Epithelioid hemangioendothelioma	1	0
	Extraskeletal myxoid chondrosarcoma	0	1
	Leiomyosarcoma	0	1
	Myxofibrosarcoma	2	0
	Rhabdomyosarcoma	1	0
	Synovial sarcoma	0	1
	Sarcoma NOS	0	1
Time since diagnosis (years)	Median (range)	8 (3–19)	6 (0–12)
Stage at study enrollment	Localized	1	1
	Metastatic disease	1	3
	Follow-up	5	9
Level of amputation	Toes/fingers/partial hand/partial foot	1	2
	Below knee/elbow (including hand/foot)	1	2
	Through knee/elbow	0	2
	Above knee/elbow	1	4
	Disarticulation	2	0
	Hindquarter/forequarter	2	3
Prosthesis	Yes	2	9
	No	4	3
	Not applicable	1	1
Dominant arm affected (in case of upper extremity sarcoma)	Yes	5	N/A
Side amputation (in case of lower extremity/pelvic sarcoma)	Right limb	N/A	7
	Left limb	N/A	6
	Pelvic	N/A	1
Other treatments^a^	Only surgery	3	7
	Radiotherapy	0	4
	Chemotherapy	3	2
	Watchful waiting for metastasis	1	0
	Isolated limb perfusion	1	1
	Isolated lung perfusion	0	1
Active treatment	Yes	3	7
Comorbid disease	None	2	5
	One	4^b^	6^d^
	Two or more	1^c^	2^e^

^a^Numbers do not add up to total *N* because multiple options are possible

^b^Diabetes, hypertension, thyroid disease, Crohn’s disease

^c^Chronic pulmonary disease and any tumor

^d^Hypertension, chronic pulmonary disease, any tumor, deep venous thrombosis, Perthes disease, thyroid disease

^e^Any tumor and coronary artery disease, atrial fibrillation and arthrosis

### ICF Categories for Physical Functioning of Patients with Sarcoma Who Underwent Amputation

Analysis of the interview data showed that, within the conceptual framework of PF of the ICF, an amputation impacts five interacting domains: body functions, body structures, structural impairments, activity and participation, and environmental factors. Body functions are defined as the physiological functions of body systems (including psychological functions). Body structures are anatomical of the body, such as organs, limbs, and their components, and are closely linked to body functions. Structural impairments are defined as problems in structure, such as a loss of (part of) limb due to amputation. Activity is defined as the execution of a task of action by individual. Participation is defined as involvement in a life situation. Activity and participation domains showed overlap and are reported together. Environmental factors are part of the physical, social, and attitudinal environment in which people live and conduct their lives.

**Fig. 1:**
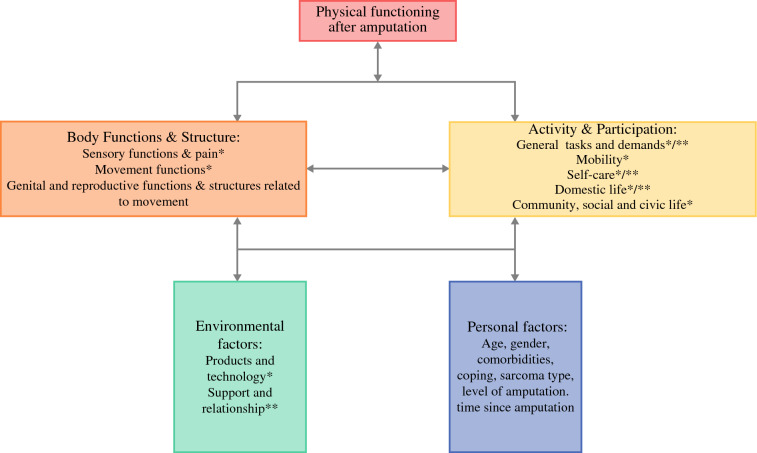
ICF categories for physical functioning after amputation; body functions are defined as the physiological functions of body systems (including psychological functions). Body structures are anatomical of the body, such as organs, limbs, and their components, and are closely linked to body functions; structural impairments are defined as problems in structure, such as loss of (part of) limb due to amputation; activity is defined as the execution of a task of action by individual; participation is defined as involvement in a life situation; activity and participation domains showed overlap and reported together; environmental factors are part of the physical, social, and attitudinal environment in which people live and conduct their lives; asterisk(s) for the environmental factors are used to indicate in what other categories of the ICF model these factors play a role for physical functioning after amputation

For an overview of the relevant ICF categories for physical functioning by level of amputation and sarcoma subtype, see Fig. 1 and Table 2. In the category body functions and structures, the following were identified: sensory functions and pain and structures related to movement, movement functions, and genital and reproductive functions and structures related to movement. In the category activity and participation, the affected areas included general tasks and demands, mobility, self-care, domestic life, and community, social, and civic life. In the category environmental factors, products and technology*, as well as support and relationship**, were included. The asterisk(s) for the environmental factors are used to indicate in what other (sub)categories of the ICF model these factors play a role for physical functioning after amputation. Most categories included patients with BS and STS, except genital and reproductive functions (only BS). However, not every patient discussed sexual problems during the interviews. The pelvic category consisted of a single bone sarcoma patient.

**Table 2:** Overview of relevant ICF categories for physical functioning by level of amputation and sarcoma subtype

ICF category/level amputation		Upper extremity amputation	Lower extremity amputation	Pelvic amputation
	**Sensory functions and pain and structures related to movement**			
		x (STS/bone)	x* (STS/bone)	
**Body functions and structures**	Sensation of pain and structure of lower extremity/ Environmental factors/products and technology*/assistive products and technology for personal indoor and outdoor mobility and transportation		Pain when walking Pain when sitting down because of prosthesis* Phantom pain	
	Sensation of falling/ Environmental factors/products and technology*/assistive products and technology for personal indoor and outdoor mobility and transportation	Fear of falling when riding bicycle	Fear of falling when riding bicycle* Fear of falling when walking down incline* Fear of falling when walking stairs*	
	**Movement functions**			
			x* (STS/bone)	
**Body functions and structures**	Gait pattern functions/ Environmental factors/products and technology*/assistive products and technology for personal indoor and outdoor mobility and transportation		Different posture with prosthesis* Changed gait with prosthesis*	
	**Genital and reproductive functions & structures related to movement**			
			x (bone)	x (bone)
**Body functions and structures**	Sexual functions and structure of the penis/structure of vagina and external genitalia/bones of pelvic region		Difficulties having sex male Difficulties having sex female	Difficulties having sex female
	**General tasks and demands**			
		x (STS*/**/bone)	x*(STS**/bone)	
**Activity and participation**	Managing one’s own activity level/ Environmental factors/support and relationship** Environmental factors/products and technology*/assistive products and technology for personal indoor and outdoor mobility and transportation	Do not want to be dependent on others**	Activities take more energy/effort/time and attention* Do not want to be dependent on others**	
	**Mobility**			
		x* (STS/bone)	x* (STS/bone)	x* (bone)
**Activity and participation**	Kneeling/ Environmental factors/products and technology*/assistive products and technology for personal indoor and outdoor mobility and transportation		Difficulties kneeling with prosthesis* Need support when getting up from kneeling or squatting*	
	Maintaining a sitting position/ Environmental factors/products and technology*/assistive products and technology for personal indoor and outdoor mobility and transportation		Difficulties sitting down	Difficulties sitting down*
	Bending/ Environmental factors/products and technology*/assistive products and technology for personal indoor and outdoor mobility and transportation		Difficulties bending with prosthesis*	
	Maintaining standing position/ Environmental factors/products and technology*/assistive products and technology for personal indoor and outdoor mobility and transportation		Difficulties standing for a long time*	
	Maintaining a body position other specified/ Environmental factors/products and technology*/assistive products and technology for personal indoor and outdoor mobility and transportation		Difficulties maintaining balance with prosthesis*	Difficulties maintaining balance with prosthesis*
	Lifting and carrying other specified/ Environmental factors/products and technology*/assistive products and technology for personal indoor and outdoor mobility and transportation	Difficulties carrying heavy items Difficulties lifting something heavy	Difficulties carrying heavy items* Difficulties carrying something when using crutches Difficulties lifting something heavy*	
	Grasping	Difficulties grasping objects		
	Other specified fine hand use	Difficulties fine hand use Difficulties writing		
	Walking short distances/ Environmental factors/products and technology*/assistive products and technology for personal indoor and outdoor mobility and transportation		Difficulties walking, takes concentration and energy*	
	Walking on different surfaces/ Environmental factors/products and technology*/assistive products and technology for personal indoor and outdoor mobility and transportation		Difficulties walking up or down incline*	
	Walking long distances/ Environmental factors/products and technology*/assistive products and technology for personal indoor and outdoor mobility and transportation		Difficulties walking long distances	Difficulties walking long distances*
	Climbing/ Environmental factors/products and technology*/assistive products and technology for personal indoor and outdoor mobility and transportation	Difficulties climbing a ladder	Difficulties climbing a ladder*	
	Running/ Environmental factors/products and technology*/assistive products and technology for personal indoor and outdoor mobility and transportation		Difficulties running	Difficulties running*
	Swimming/ Environmental factors/products and technology*/assistive products and technology for personal indoor and outdoor mobility and transportation		Difficulties swimming*	
	Going up and down the stairs/ Environmental factors/products and technology*/assistive products and technology for personal indoor and outdoor mobility and transportation		Difficulties walking stairs*	
	Moving around using equipment/ Environmental factors/products and technology*/assistive products and technology for personal indoor and outdoor mobility and transportation	Use an e-bike* Use a cargo bike*	Difficulties riding a bicycle* Use a walker* Use a wheelchair* Use insoles in shoes* Use special shoes* Use of triple chair* Use an e-bike* Use a stair lift* Use handrails*	Use a wheelchair Use prosthesis for balance on hand bike* Use a hand bike
	Driving	Difficulties driving a car		
	**Self-care**			
		x (STS/bone)	x* (STS/bone**)	x* (bone)
**Activity and participation**	Dressing/putting on clothes and Other specified washing oneself/ Environmental factors/support and relationship**/immediate family Environmental factors/products and technology*/assistive products and technology for personal indoor and outdoor mobility and transportation	Difficulties closing buttons Difficulties putting on a bra	Difficulties with dressing and showering**	Difficulties with dressing*
	Putting on footwear	Difficulties tying shoelaces		
	Other specified washing oneself/ Environmental factors/products and technology*/assistive products and technology for personal indoor and outdoor mobility and transportation		Use a bath plank* Use handrails to shower* Use a chair to shower* Use special prosthesis in shower*	Use handrails to shower*
	Caring for hair	Difficulties styling my hair		
	Other specified toileting/ Environmental factors/products and technology*/assistive products and technology for personal indoor and outdoor mobility and transportation		Using handrails for toilet* Using urinal in night*	Using handrails for toilet* Removing prosthesis before toileting*
	**Domestic life**			
		x (STS**/bone)	x* (STS/bone**)	x** (bone)
**Activity and participation**	Household tasks/ Environmental factors/support and relationship**/immediate family Environmental factors/products and technology*/assistive products and technology for personal indoor and outdoor mobility and transportation	Difficulties working in the garden** Difficulties ironing clothes** Difficulties changing bed sheets** Difficulties preparing and serving food and drinks**	Difficulties ironing** Difficulties working in the garden* Difficulties preparing and serving food or drinks**	Difficulties with vacuuming* Difficulties preparing and serving food and drinks**
	Assisting others with self-care/ Environmental factors/products and technology*/assistive products and technology for personal indoor and outdoor mobility and transportation	Difficulties caring for (grand)children	Difficulties caring for (grand)children*	
	**Community, social, and civic life**			
			x* (STS/bone)	x (bone*)
**Activity and participation**	Sports/ Environmental factors/products and technology*/assistive products and technology for personal indoor and outdoor mobility and transportation		Difficulties kickboxing* Difficulties mountain biking*	Difficulties with dancing*
**Environmental factors**				
	Products and technology*	x (STS/bone)	x (STS/bone)	x (bone)
	**Community, social, and civic life**	x (STS)	x (STS/bone)	x (bone)

Body functions are defined as the physiological functions of body systems (including psychological functions). Body structures are anatomical of the body, such as organs, limbs, and their components, and are closely linked to body functions. Structural impairments are defined as problems in structure, such as loss of (part of) limb due to amputation. Activity is defined as the execution of a task of action by individual. Participation is defined as involvement in a life situation. Activity and participation domains showed overlap and reported together. Environmental factors are part of the physical, social, and attitudinal environment in which people live and conduct their lives.

### ICF Subcategories of Body Functions and Structures and Themes for Physical Functioning of Patients with Sarcoma Who Underwent Amputation

In Table S2, the subcategories, themes, and quotes of body functions and structures are presented. The overlapping subcategories between upper and lower extremity are underlined. Lower extremity patients and one patient with pelvic sarcoma reported that their PF related to body functions and structures was limited in the following areas: sensation of pain and structure of lower extremity*, sensation of falling*, gait pattern functions*, sexual functions and structure of penis/structure of vagina and external genitalia, and bones of pelvic region. For upper extremity amputation, the singular theme sensation of falling is highlighted. This is an example of the fear of falling when riding a bicycle after upper extremity amputation: “But still riding with just one hand, of course. I said, ‘What did you expect? When you’re cycling through the woods and hit a pine cone or some branches, sometimes you can barely stay upright even with both hands on the handlebars” [P2, female, 60–70, STS, disarticulation amputation, no prosthesis].

The following quote illustrates the sensation of falling when riding a bicycle with a prosthesis after lower limb amputation: “And I don’t cycle anymore, haven’t for about five years. I just don’t dare anymore. Because in the year before my surgery and the two years prior to that, I fell off my bike once every year. I did learn to cycle with the prosthesis during rehabilitation, but I just find the traffic too busy, too unpredictable, and I’m afraid I’ll fall. So, I don’t cycle anymore, I just don’t do it” [P37, female, 70–80, STS, above knee amputation, prosthesis].

### ICF Subcategories of Activities and Participation and Themes of Physical Functioning of Patients with Sarcoma Who Underwent an Amputation

In Table S3, the subcategories, themes, and quotes of activities and participation related to physical functioning are presented. After upper extremity amputation, patients experienced difficulties with physical functioning in the following areas: managing one’s own activity level**, lifting and carrying (other specified), grasping, other specified hand use, climbing, moving around using equipment**, moving around using transportation, dressing/putting on clothes, putting on footwear, caring for hair, household tasks, and assisting others with self-care. This is an example of one of the household tasks “preparing and serving food and drinks” after an upper extremity amputation: “And serving. Yeah, my husband usually takes it off the stove and brings it inside. He says, ‘I’d rather do it myself than something will happen.’ But sometimes I do put a pot on the table myself. And if it’s a pot with a handle, that’s much easier. (…) Yeah, like a wok pan. I’ve also seen, I’ve found some pans that you normally use with two handles, but this one has a single handle” [P2, female, 60–70, STS, disarticulation amputation, no prosthesis].

For lower extremity amputation and one patient with pelvic amputation, more and overlapping subcategories (underlined) with upper extremity amputation were reported related to physical functioning: managing one’s own activity level**, kneeling*, maintaining sitting position*, bending, maintaining standing position, maintaining body position (other specified)*, lifting and carrying (other specified)*, walking short distances*, walking on different surfaces, walking long distances*, climbing, running, swimming*, going up and down the stairs, moving around using equipment*, driving, dressing/putting on clothes, (other specified) washing oneself*, (other specified) toileting*, household tasks, shopping, assisting others with self-care*, and sports. The following quotes are two examples of the household task “preparing and serving food and drinks” after lower extremity and pelvic amputation:

“I can possibly do that while sitting, and serving… well, serving... To give a very practical example, I don’t take a casserole dish out of the oven. I leave that to my wife because I’m less stable when standing. Then my wife pushes me aside and says, ‘I’ll do it for you” [P38, male, 60–70, bone, above knee amputation, prosthesis]; and “Cooking is indeed difficult for me, and serving is something I definitely can’t do. I can’t even carry my own cup of coffee” [P1, female, 50–60, bone hindquarter amputation, prosthesis].

Furthermore, patients made general remarks on making adjustments to do certain activities and how activities require more energy, time, or attention to complete than before amputation. Patients also mentioned that they would avoid certain tasks that they cannot do anymore since their amputation. For many of the identified limited activities, patients mentioned that either a prosthesis or another assistive aid (i.e., wheelchair or crutch) enabled them to complete the activity, although in some cases, the prosthesis itself limited the activity.

## Discussion

Amputation due to sarcoma has a profound and multifaceted impact on physical functioning (PF), as illustrated by our qualitative findings. Participants with upper (*n* = 7), lower (*n* = 12), or pelvic (*n* = 1) extremity sarcoma described a wide range of experiences when responding to the C30 and TESS, often shaped by the use of prosthesis or other assistive devices, the level and location of the amputation, and the availability of support. These contextual factors influenced how patients interpreted and answered questions about physical functioning, leading to inconsistencies in how physical functioning is reported and understood. Importantly, neither the C30 nor the TESS explicitly accounts for these contextual factors, which can lead to inconsistent or inaccurate scoring of physical functioning in this population.

By applying the ICF framework, we were able to map these experiences across multiple domains, including body functions and structures (e.g., phantom pain, sexual function), activities and participation (e.g., dressing, walking, preparing meals), and environmental factors (e.g., assistive devices, social support). This holistic perspective revealed that neither the C30 nor the TESS sufficiently captures the complexity of physical functioning in this population. Both instruments lack explicit guidance on how to report physical functioning when assistive devices are used, and they omit key domains such as environmental support and pain-related limitations.

Our findings are consistent with previous research. Blight et al. emphasized the need for improved PROMs tailored to the functional diversity within extremity sarcoma populations.^37^ Hoftiezer et al. reported significantly lower TESS scores in amputees compared with patients who underwent LSS, suggesting that current PROMs may not adequately reflect the experiences of all patients with sarcoma.^38^ Sions et al. similarly emphasized the need for PROMs that address prosthesis use and stump pain, factors that were central to our participants’ narratives.^39^ These studies, together with our findings, point to a gap between what is measured and what matters to patients.

Van Eck et al. identified common task-related difficulties among upper and lower extremity amputees, such as dressing, preparing food, walking on uneven surfaces, and engaging in sports.^40^ These challenges were echoed in our interviews and can be directly linked to ICF categories, reinforcing their clinical relevance. However, Van Eck et al. did not explore the influence of contextual factors such as prosthesis use or assistive devices, which were central to our participants’ experiences.^40^ Similarly, phantom pain, mentioned by our participants, remains unaddressed in both the TESS and C30, despite its significant impact on daily functioning.

Vetchy et al. further highlighted the complexity of physical functioning after amputation, noting limitations in mobility, as well as the burden of phantom limb pain and residual limb discomfort.^41^ Although their study did not evaluate the C30 or TESS directly, their findings mirror those of our participants, particularly regarding the variability in physical functioning depending on whether prosthesis or assistive devices are used. These insights underscore the need to refine existing PROMs to better reflect the lived experiences of amputees.

Taken together, our findings and those from prior studies underscore the need for a more comprehensive, context-sensitive approach to assessing physical functioning in patients with sarcoma after amputation. Current PROMs such as the C30 and TESS fall short in capturing the full range of challenges faced by this population, particularly when assistive devices, environmental support, and pain are involved. To improve the validity and clinical utility of these measures, future research should explore strategies such as: providing clear instructions on how to complete PROMs (e.g., whether to report physical functioning with or without assistive devices), including complementary items that address context-specific challenges (e.g., a new question about phantom pain or two questions about prosthesis fit in the recently developed Sarcoma Assessment Measure),^42^ and supplementing PROMs with clinical interviews or case report forms (CRFs) to capture nuanced information. Further research is needed to determine which of these approaches—or combination thereof—provides the most accurate, feasible, and patient-centered solution. A study validating the Patient-Reported Outcome Measure Information System (PROMIS) in patients with sarcoma undergoing surgery, compared with the current gold standard TESS (clinicaltrials.gov NCT07227961),^43^ together with the current EORTC VOICE study on relevance and importance of sarcoma-subgroup specific issues, will provide guidance on the optimal measurement strategy for patients with sarcoma who underwent amputation.^32^

Finally, timely preoperative referral to rehabilitation specialists is essential. Oncologists and surgeons need to ask for context (prosthesis use and fit, assistive devices, or help from others) when interpreting the TESS and C30 scores. Where appropriate, referral to sexologists may also be considered, particularly when the location or nature of the amputation is likely to affect body image, intimacy, or sexual well-being. Such referrals may help patients better manage expectations and prepare for postoperative challenges in a holistic and person-centered manner.

### Strengths and Limitations

This study’s strengths include the inclusion of a diverse group of participants, varying in age, gender, tumor type, amputation level, and treatment experiences, which enhances the depth and relevance of the findings. The application of the ICF framework provided a systematic structure for categorizing functional impairments, facilitating comparison with broader health models. However, there are limitations. Although we aimed for diversity in amputation levels using a stratification matrix, some subgroups were underrepresented or absent. Specifically, only one case was included for pelvic amputation and through-elbow upper extremity amputation, and no cases for lower extremity disarticulation. Upper extremity amputations (fingers/partial hand and below elbow) were also limited due to rarity and fewer reported issues. These gaps may restrict the transferability of findings to these specific subgroups. All participants were recruited in the Netherlands with specific cultural and healthcare context (prosthesis access, rehabilitation resources) during the COVID-19 pandemic, which might have resulted in pandemic-related lifestyle changes. Another limitation is the wide range of time since diagnosis, as the experience of physical functioning after amputation may change over time and patients may find their own solutions, potentially also limiting the transferability of the findings.

## Conclusions

This study highlights the need for a tailored strategy to assess physical functioning in patients with sarcoma following amputation. The C30 and TESS currently lack guidance on responding with prostheses, assistive devices, or support from others, which can undermine the validity of results. Future research should explore strategies to enhance the interpretation of PROM scores in this population, such as clear instructions for completing the questionnaires on the basis of individual functional circumstances. A more nuanced and context-sensitive approach will improve the accuracy and clinical utility of PROMs in sarcoma survivorship care and research.

## Supplementary Information

Below is the link to the electronic supplementary material.

Supplementary file1 (DOCX 44 KB)
