# Supplementary material for: Understanding Physical Functioning in Patients with Sarcoma After Amputation: Using an International Classification of Functioning, Disability and Health (ICF) Approach
Source: Ann Surg Oncol. 2026 Apr 5;33(7):6867–80. doi: 10.1245/s10434-026-19373-y (PMC13242479; doi:10.1245/s10434-026-19373-y)
Supplement: Supplementary file 1 — Supplementary file1 (DOCX 44 KB) [file 10434_2026_19373_MOESM1_ESM.docx]

**Supplementary Materials:**

S1: interview guide

**Phase 1A - Patient Interview Script:**

**Outline**

- Introduce the study and the reasons we are asking them to participate
- Assure them about maintaining confidentiality
- Explain the need to gather some personal information about them for the research study
- Ask them first about current issues/ experiences they are having because of having been diagnosed with sarcoma
- Explain what we expect them to rate today *(i.e. the EORTC QLQ-C30 and TESS (upper/lower extremity)*
- Present EORTC QLQ-C30 and TESS (upper/lower extremity) and ask them to rate it *(explain EORTC QLQ-C30 is a cancer QOL questionnaire for patients under treatment and TESS is a physical functioning questionnaire for patients undergoing surgery for sarcoma of the extremities)*
- Ask them to tell about any issues they feel are missing from the questionnaires
- Thank them for sharing their experiences with us and providing feedback on our questionnaire

**PHASE 1:**

**GENERATION OF RELEVANT HEALTH-RELATED QUALITY OF LIFE ISSUES IN SARCOMA PATIENTS WHO UNDERWENT (EXTREMITY) AMPUTATION *(RED FONT INDICATES INTERVIEWER’S SCRIPT****)*

The study coordinator should begin the interview with some introductory remarks to explain the nature and purpose of the research study. For example:

- Introduce the study and the reasons we are asking them to participate

***We are asking for your help in devising a questionnaire which will be used to monitor the experiences of people who have been diagnosed with sarcoma in an extremity and underwent amputation. I would like to ask you a few things about your health and functioning.***

- Assure them about maintaining confidentiality

***Remember all of this information you are providing will remain confidential and your data will be assigned a unique research number so your name will not appear anywhere these data are used in the future.***

- Explain the need to gather some personal information about them for the research study

***In order to provide some background information for our research I would like to gather some personal information about your life circumstances including current work and relationship status.*** (Use an appropriate list to complete with the patient)

- Ask them first about their (extremity) amputation after being diagnosed with sarcoma, but keep this brief

***Can you tell me about the (extremity) amputation you underwent because of being diagnosed with sarcoma?***

- Ask them about current issues/ experiences they are having because of the (extremity) amputation after being diagnosed with sarcoma

***What are the most important things you experienced? Or can you think of any additional experiences? Do you wear a prosthesis (type and frequency of use?), sling or brace (frequency of use?)? Do you use a walking aid (type and frequency of use?) or a wheelchair (frequency of use?) or other supports (type and frequency of use?)? What is your dominant arm/hand (i.e. in case of amputation arm/hand) and what is the impact on functioning?***

***Follow-up questions***

The use of follow-up questions or "probes" will be required in the majority of interviews. The appropriate wording is dependent on the topic at hand, but should always be in an open, nonjudgmental way. For example:

· If the answer is too general and indefinite, the follow-up may be

***In what way? Just how do you mean? Can you give me an example?***

· If the answer is incomplete, the questions may be:

***Any other reasons? Would you tell me a little more about that?***

It may be useful to prompt the patient to consider specific domains, especially if the literature review has suggested that these may be relevant to the patient group. Some examples are shown:

**Major questions**

**· *Physical functioning is doing or being able to do a variety of physical activities such as bathing, dressing, walking, bending, climbing stairs or running. Could you tell us about your physical functioning? (e.g. lower extremity: external supports required, walking ability, gait alterations; upper extremity: hand positioning (i.e. actively position hand for function activities), manual dexterity (i.e. complex functions hand; pinch or grasp) and lifting ability (i.e. lift or place objects unassisted))***

**· *What are you not able to do that you would formerly do before your illness, and why?***

**· *Are you limited in normal daily activities (e.g.- work, household, shopping, taking care of the children, sports) compared to before your illness? What is it that limits you (e.g. pain and emotional acceptance)?***

**Minor questions**

**· *Are you undertaking fewer social activities (e.g.-hobbies, meeting up with friends) and why?***

**· *Have changes in relationships with family/friends occurred?***

**· *Do you have financial problems or worries due to your illness (e.g. insurance, mortgage, loans)?***

**· *Have your personal feelings changed (e.g.-satisfaction with life, spirituality)?***

**· *Has your emotional wellbeing changed (e.g.-feelings of anxiety or worrying)?***

**· *Are there any other issues or comments you would like to make regarding your illness and treatment and your quality of life?***

- Explain what we expect them to rate today *(i.e. the QLQ C-30 and the TESS (upper/lower extremity)*

***Today we are asking you to rate two questionnaires and provide us with your feedback about them.***

- ***The QLQ C-30 is an existing questionnaire used for patients under treatment for many different types of cancer. The TESS (upper/lower extremity) is an existing questionnaire used for extremity sarcoma patients who underwent surgery.***
- Present QLQ-C30 and TESS (upper/lower extremity) and ask them to rate it *(explain QLQ-C30 is a cancer QOL questionnaire for patients under treatment and TESS (upper/lower extremity) is a physical functioning questionnaire for extremity sarcoma patients undergoing surgery)*

***The QLQ-C30 is an existing questionnaire that asks about patients under treatment for cancer and their quality of life. These questions may also be of value for patients with sarcoma in an extremity who underwent amputation. The TESS (upper/lower extremity) is an existing questionnaire that asks about patients undergoing surgery of upper or lower extremities for sarcoma and their physical functioning.***

***Today we would like you to rate these questions according to ‘Relevance’ .***

***“Relevance” refers to the frequency with which a specific issue occurs and, when it occurs, the trouble it may cause. Thus, the more frequently an issue occurs and the more trouble it causes, the more relevant it will be.***

***Could you please indicate for each question separately the extent to which you find it relevant for you?***

1. ***Not at all relevant***
2. ***A little relevant***
3. ***Quite relevant***
4. ***Very relevant***

**(Place the QLQ-C30 and the TESS (upper/lower extremity) before the patient who rates it)**

- Ask them to tell us about any issues they feel are missing from the questionnaires

***Now that you have completed both these questionnaires can you please tell whether you think these questionnaires describe sufficiently your current health and physical functioning? If no: please name the issues you think need to be changed or added so it will be able to describe your physical functioning?***

***Relevance: Why do you consider these question(s) not at all or only a little relevant for evaluating your physical functioning? (score 1-2)***

***Breath of coverage: Can you tell me about any issues you feel are missing related to the experiences of physical functioning you may have had as a result of being diagnosed with sarcoma in an extremity and underwent amputation which you feel has affected your quality of life, in both a positive or negative way.***

- Ask them to tell us about the interpretation and wording of questions and response options from the questionnaires

***What is your interpretation of the questions of these questionnaires (specify: QLQ-C30/TESS upper/lower extremity)? How would you fill in these questions? (if applicable: with/without wearing prosthesis, sling, brace; with/without using walking aid/wheelchair)***

***Appropriateness: What questions or response options did you perceive as inappropriate or upsetting? How would you suggest to improve the wording of the questions or response options (e.g. not applicable, impossible to do)?***

***Additional question if there is sufficient time:***

***Relative importance: What is the difference between the two questionnaires? Please select the questionnaire (and questions) that is/are most appropriate when evaluating your physical functioning after amputation? Why?***

***Follow-up questions***

·The interviewer may use additional neutral probes, e.g*.:*

**·*Can you tell me more about that? Can you think of additional experiences?***

· Other follow-ups could ask:

**·*What makes you think this? What was there in the question that made you feel that way?*"**

It may be useful to prompt the patient to consider specific domains, especially if the literature review has suggested that these may be relevant to the patient group. Some examples are shown:

**·** ***Do you have any other symptoms not mentioned in the questionnaire?***

**· *Do you have other problems with your physical functioning?***

**· *Do you experience pain that affects your physical functioning? Do you use pain medication or other equivalent measures to relief the pain (type and frequency of use)?***

**· *How is your emotional reaction to or perception of the functional result of the extremity amputation? Would you do it again or recommend it to others?***

- Thank them for sharing their experiences with us and providing feedback on our questionnaire

***Thank you very much for your time today and for agreeing to participate in this research study. Your personal information and experience is very valuable to us in the development of this Sarcoma questionnaire.***

**Table S2 Body functions & structures**

| **Sensory functions and pain (ICF category)** | [**ICF code**](https://icd.who.int/browse/2025-01/icf/en)**s** | **Upper extremity amputation**  **Theme** *quote* [study number, gender, age range, sarcoma type, amputation level, (no) prosthesis] | **Lower extremity amputation**  **Theme** *quote* [study number, gender, age range, sarcoma type, amputation level, (no) prosthesis] | **Pelvic amputation**  **Theme** *quot*e [study number, gender, age range, sarcoma type, amputation level, (no) prosthesis] |
| --- | --- | --- | --- | --- |
| **Sensation of pain & structure of lower extremity/**  **Environmental factors/products and technology*/**  **assistive products and technology for personal indoor and outdoor mobility and transportation** | **b280 &**  **s750/**  **e1201** |  | **Pain when walking:** *“But that surgery didn’t help. To this day, I still have severe pain when walking”* [P33, female, 40-50, STS, toes/partial foot amputation, no prosthesis].  **Pain when sitting down because of prosthesis***:  *"When you have a socket prosthesis, whether it’s a vacuum type or another, there’s always something poking into your buttock, so sitting just isn’t comfortable. You want to sit because you’re tired, but you also don’t really want to sit, because then you’ll start getting pain in your buttocks again”* [P30, female, 30-40, bone, above knee amputation, prosthesis].  **Phantom pain:**  *"I do experience phantom pain, but also pain from sitting too long—like in my back, it just becomes heavy. But I’ve had so much pain over the years, I can’t spend my whole day feeling it.."* [P34, female, 30-40, bone, hindquarter amputation no prosthesis] |  |
| **Sensation of falling/**  **Environmental factors/products and technology*/assistive products and technology for personal indoor and outdoor mobility and transportation** | **b2402/**  **e1201** | **Fear of falling when riding bicycle:** *"That also has to do with cycling, which I just can’t do. You just have a limitation. And they want me to get back on a two-wheeler, but I just don’t see that happening."* [P2, female, 60-70, STS, disarticulation amputation, no prosthesis] | **Fear of falling when riding bicycle*:**  *"And I don’t cycle anymore, haven’t for about five years. I just don’t dare anymore. Because in the year before my surgery and the two years prior to that, I fell off my bike once every year. I did learn to cycle with the prosthesis during rehabilitation, but I just find the traffic too busy, too unpredictable, and I’m afraid I’ll fall. So, I don’t cycle anymore, I just don’t do it"* [P37, female, 70-80 STS, above knee amputation, prosthesis].  **Fear of falling over when walking down incline*:**  *"No, because that ankle doesn’t move, it’s stiff. If you can’t place your foot properly and can’t roll through it well, I feel like I’m going to fall forward or something. I just find that really difficult."* [P38, male, 60-70, bone, above knee amputation, prosthesis]  **Fear of falling when walking stairs*:**  *"I can climb stairs on crutches. So if you’re in a big building and there are steps up, like at a station or something, I can do all that. But yes, there is a higher risk of falling."* [P3, male, 30-40 bone, hindquarter amputation, no prosthesis] |  |
| **Movement functions (ICF category)** | [**ICF codes**](https://icd.who.int/browse/2025-01/icf/en) | **Upper extremity amputation**  **Theme** *quote* [study number, gender, age range, sarcoma type, amputation level, (no) prosthesis] | **Lower extremity amputation**  **Theme** *quote* [study number, gender, age range, sarcoma type, amputation level, (no) prosthesis] | **Pelvic amputation**  **Theme** *quote* [study number, gender, age range, sarcoma type, amputation level, (no) prosthesis] |
| **Gait pattern functions**  **Environmental factors/products and technology*/assistive products and technology for personal indoor and outdoor mobility and transportation** | **b770/**  **e1201** |  | **Different posture with prosthesis*:**  *"(…) I can now see that my posture is different from when I had two normal legs. (…)”* [P19, male, 50-60, STS, below knee amputation, prosthesis]  **Changed gait with prosthesis*:** *"It’s of course slower than normal. And the stride length is also shorter. It may not make a huge difference, but it does make a difference. And the pace is of course different."* [P20, male, 80-90, STS, through knee amputation, prosthesis] |  |
| **Genital and reproductive functions (ICF category)** | **ICF code** | **Upper extremity amputation**  **Theme** *quote* [study number, gender, age range, sarcoma type, amputation level, (no) prosthesis] | **Lower extremity amputation**  **Theme** *quote* [study number, gender, age range, sarcoma type, amputation level, (no) prosthesis] | **Pelvic amputation**  **Theme** *quote* [study number, gender, age range, sarcoma type, amputation level, (no) prosthesis] |
| **Sexual functions &**  **structure of the penis/**  **structure of vagina and external genitalia/**  **bones of pelvic region** | **b640 &**  **s6305/**  **s6303/**  **s74000** |  | **Difficulties having sex:**  *Patient: "Because it’s just broken.* *My sex life is broken. I wasn’t told that beforehand. Researcher: You didn’t know?” Patient: “No, I asked the doctor, 'Will I still be able to have an erection?' 'Yes, you’ll still be able to get an erection.' Researcher: Oh, he said it would still be possible? Patient: “Yes, and then when it came down to it, it didn’t happen. And then he said again, 'No, it makes sense that it doesn’t work.' Okay then, jerk. We also went to a sexologist and she said, 'Yes, that’s because a lot is missing on one side, including muscles and other things.'* *That just leads to a poor erection. You can still have sexual activity in other ways, but traditionally you can no longer have intercourse, that’s no longer possible."* [P3, male, 30-40, bone, hindquarter amputation, no prosthesis]  **Difficulties having sex female:** *"Well, I think it could all be possible, but I had all that edema in my labia, which hasn’t been very pleasant."* [P34, female, 30-40, bone, hindquarter amputation no prosthesis] | **Difficulties having sex**: *"Now, I may still be the same woman, but physically, I’m not anymore. And on top of that, I’m also unlucky enough to have the highest level of amputation, and the bone that was put back to create a kind of sitting bone isn’t positioned correctly, so my feminine side, so to speak, is closed off. So, you can imagine what I mean. So yes, a lot changes, and that’s really difficult."* [P1, female, 50-60, bone, hindquarter amputation, prosthesis] |

**Table S3 Activity and Participation**

| **General tasks and demands (ICF category)** | [**ICF codes**](https://icd.who.int/browse/2025-01/icf/en) | **Upper extremity amputation**  **Theme** *quote* [study number, gender, age range, sarcoma type, amputation level, (no) prosthesis] | **Lower extremity amputation**  **Theme** *quote* [study number, gender, age range, sarcoma type, amputation level, (no) prosthesis] | **Pelvic amputation**  **Theme** *quote* [study number, gender, age range, sarcoma type, amputation level, (no) prosthesis] |
| --- | --- | --- | --- | --- |
| **Managing one’s own activity level/**  **Environmental factors/Support and relationship****  **Environmental factors/products and technology*/assistive products and technology for personal indoor and outdoor mobility and transportation** | **d203/**  **e399/**  **e1201** | **Don’t want to be dependent on others**:**  *"I just really need things to be adjusted as quickly as possible so I can get on with my life. I simply don’t want to be continuously dependent on my husband. At certain times, my husband is a caregiver, but where it’s not necessary, I want to do things myself. I just handle it.*" [P2, female, 60-70, STS, disarticulation amputation, no prosthesis] | **Activities take more energy/effort/time and attention***:  *"Look, I can't do what you do anymore. For example, if you drop something. You're writing, and your pen falls on the floor, it takes a few minutes before you can pick it up. You have to bend down, stretch your leg."*  [P20, male, 80-90STS, through knee amputation, prosthesis]  **Don’t want to be dependent on others**:**  *"But it’s not my style to be dependent, so I sometimes struggle with that. And I’m not fully dependent, I would really find that awful. But if you were, your relationship would also change, and that would be very difficult."* [P37, female, 70-80, STS, above knee amputation, prosthesis] |  |
| **Mobility (ICF category)** | [**ICF codes**](https://icd.who.int/browse/2025-01/icf/en) | **Upper extremity amputation**  **Theme** *quote* [study number, gender, age range, sarcoma type, amputation level, (no) prosthesis] | **Lower extremity amputation**  **Theme** *quote* [study number, gender, age range, sarcoma type, amputation level, (no) prosthesis] | **Pelvic amputation**  **Theme** *quote* [study number, gender, age range, sarcoma type, amputation level, (no) prosthesis] |
| **Kneeling/**  **Environmental factors/products and technology*/assistive products and technology for personal indoor and outdoor mobility and transportation** | **d4102/**  **e1201** |  | **Difficulties kneeling with prosthesis*:**  *With my left leg, I can't bend my knee, so I have to throw my left leg completely out of the way, almost like you have a stiff leg. I have to move it completely aside, and then I can bend down."* [P38, male, 60-70, bone, above knee amputation, prosthesis]  **Need support when getting up from kneeling or squatting*:**  *"It’s relevant, but I always need a tool to make it happen. So, if I were to fall on the street, someone would need to offer me a chair or something, and then I could stand up. But they shouldn’t try to pull me or anything, that doesn’t work. I need to grab something and rest my arms on it so I can use my arms to push myself up. So, it’s relevant, but it doesn’t work well."* [P37, female, 70-80, STS, above knee amputation, prosthesis] |  |
| **Maintaining a sitting position/**  **Environmental factors/products and technology*/assistive products and technology for personal indoor and outdoor mobility and transportation** | **d4103/**  **e1201** |  | **Difficulties sitting down:**  *"I find it hard to sit for long periods. Yeah, more for your back. Because I’m really sitting on one leg, I often end up sitting on my hands or on the supports of my chair. It’s just to maintain balance in your back."* [P34, female, 30-40, bone, hindquarter amputation, no prosthesis] | **Difficulties sitting down*:**  *"And I always need cushions to sit. I tend to tip over a little bit, you know, if I'm sitting without support or something."* [P1, female, 50-60, bone, hindquarter amputation, prosthesis] |
| **Bending/**  **Environmental factors/products and technology*/assistive products and technology for personal indoor and outdoor mobility and transportation** | **d4105/**  **e1201** |  | **Difficulties bending with prosthesis*:**  *"Yeah, you asked that question earlier. I can bend down, but at some point, I have to hold my leg in a really awkward position."* [P38, male, 60-70, bone, above knee amputation, prosthesis] |  |
| **Maintaining standing position**  **Environmental factors/products and technology*/assistive products and technology for personal indoor and outdoor mobility and transportation** | **d4154**  **e1201** |  | **Difficulties standing for a long time*:**  *"I’ve become less able to do that. But it’s also heavy because the household chores themselves... Vacuuming is still doable because I just stand behind the vacuum and can move back and forth a little. But with things where you have to move left, right, forward, and backward, and find balance, it really becomes heavy. I can just feel how incredibly exhausting it is. It’s not the work itself, but just continuously standing, searching for balance with one foot. That’s really tiring."* [P38, male, 60-70, bone, above knee amputation, prosthesis] |  |
| **Maintaining a body position other specified/**  **Environmental factors/products and technology*/assistive products and technology for personal indoor and outdoor mobility and transportation** | **d4158/**  **e1201** |  | **Difficulties maintaining balance with prosthesis*:** *You're trying to maintain your balance, especially when you're standing on the prosthetic leg, trying to get your good foot into the leg of the underwear."* [P36, male, 70-80, bone, above knee amputation, prosthesis] | **Difficulties maintaining balance with prosthesis*:** *"With that prosthesis, I’m very unstable, so to speak."* [P1, female, 50-60, bone, hindquarter amputation, prosthesis] |
| **Lifting and carrying other specified/**  **Environmental factors/products and technology*/assistive products and technology for personal indoor and outdoor mobility and transportation** | **d4308/**  **e1201** | **Difficulties carrying heavy items:**  *"Yes, I can carry a heavy shopping bag, but it's difficult because I'm missing a finger, and I overstrain… I can show you; I overstrain my index finger. It bends, and it gets much more strained because it compensates for the missing finger. So, I can't just carry a really heavy shopping bag on my right side."* [P5, female, 60-70, bone, fingers/partial hand amputation, no prosthesis]  **Difficulties lifting something heavy:**  *"Like lifting a large, heavy object, say a box that's 60 by 60 and weighs 10 kilos—I just can’t lift that off the ground. It’s just not going to work."* [P29, male, 30-40, bone, forequarter amputation no prosthesis] | **Difficulties carrying heavy items*:**  *"Look, my husband carries the groceries inside, so to speak, if I’ve done the shopping. He brings them inside."* [P37, female, 70-80, STS, above knee amputation, prosthesis]  **Difficulties carrying something when using crutches:**  *"It's only difficult if you want to carry something, like a backpack or something. If it's just a bag, it swings back and forth and bumps against your legs, which is annoying."*  [P3, male, 30-40, bone, hindquarter amputation, no prosthesis].  **Difficulties lifting something heavy*:**  *"(...) but if I have to lift heavy things, it’s not convenient, because I can’t do it in a normal way, so to speak (...)"* [P30, female, 30-40, bone, above knee amputation, prosthesis]. |  |
| **Grasping** | **d4401** | **Difficulties grasping objects:**  *"If I pick up a cup or, let’s say, a fry, it slips through because I’m missing a finger. Sometimes something slips through my hand when I try to grab it, as I don’t have quite enough grip. So my gripping function is somewhat weaker. But it doesn’t really hinder me in my general, daily life with everything."* [P5, female, 60-70, bone, fingers/partial hand amputation, no prosthesis] |  |  |
| **Other specified fine hand use** | **d4408** | **Difficulties fine hand use:** *"Finer things are usually the hardest, because in the beginning, I could still manage to open a bread bag, that plastic tie around it. I figured out how to do that. It’s really small things you normally don’t even think about. The most difficult things."* [P21, male, 20-30, STS, below elbow amputation, prosthesis]  **Difficulties writing:**  *"Yes, also, for example, writing or something like that, you can still do pretty quickly… I had to learn to write with my left hand, but you also notice pretty quickly that the paper moves. You can’t stop that with your right hand, for example. So, often I look for something heavy to put on the paper, and then it works pretty well. But I must say, I don’t write whole pages, because you quickly notice that your arm just gets tired."* [P7, female, 40-50, bone, disarticulation amputation, no prosthesis] |  |  |
| **Walking short distances/**  **Environmental factors/products and technology*/assistive products and technology for personal indoor and outdoor mobility and transportation** | **d4500/**  **e1201** |  | **Difficulties walking takes concentration and energy*:**  *Patient: "Because, look, my knee doesn’t move anymore, because I only have half a knee. I have a prosthetic knee, so to speak. And dealing with that, you really have to concentrate.” Researcher: “Definitely. Because it takes energy?” Patient: “Energy and thinking carefully."* [P20, male, 80-90, STS, through knee amputation, prosthesis] |  |
| **Walking on different surfaces**  **Environmental factors/products and technology*/assistive products and technology for personal indoor and outdoor mobility and transportation** | **d4502/**  **e1201** |  | **Difficulties walking up or down incline*:**  *"As long as it’s flat, it’s fine. But if it’s a slope or something, then it’s really... It becomes really difficult. It almost becomes disastrous, I would say. Then I really don’t like it anymore because I can’t place my foot properly."* [P38, male, 60-70, bone, above knee amputation, prosthesis] |  |
| **Walking long distances/**  **Environmental factors/products and technology*/assistive products and technology for personal indoor and outdoor mobility and transportation** | **d4501/**  **e1201** |  | **Difficulties walking long distances:**  *"Or going for a walk like others, that’s really not possible. Look, I do go when my wife and kids take a walk, I always join with the crutches, but I’m basically doing top-level sports the whole time. It’s really tough. Look, and when I say, 'I’ll go in the wheelchair,' I do that sometimes, but it’s not comfortable either. Sometimes it’s necessary, but it’s not everything."*[P3, male, 30-40, bone, hindquarter amputation, no prosthesis] | **Difficulties walking long distances*:**  *"But the maximum I can manage is a little, 2 kilometers is already a lot, if I even manage it. Well, walking was one of my hobbies. We’ve always had a dog, and now we have a puppy in the house. You know, I used to spend hours in the woods, but I just can't do that anymore."*[P1, female, 60-70, bone, hindquarter amputation, prosthesis] |
| **Climbing**  **Environmental factors/products and technology*/assistive products and technology for personal indoor and outdoor mobility and transportation** | **d4551/**  **e1201** | **Difficulties climbing a ladder:**  *"My wife doesn’t let me climb the ladder at all.*"[P4, male, 70-80, STS, forequarter amputation, no prosthesis] | **Difficulties climbing a ladder*:**  *"Well, no. You know, I can't stand on a ladder to clean the windows."* [P37, female, 70-80, STS, above knee amputation, prosthesis] |  |
| **Running**  **Environmental factors/products and technology*/assistive products and technology for personal indoor and outdoor mobility and transportation** | **d4552**  **e1201** |  | **Difficulties running:**  *"Well, other than running, which I was never really a fan of, with those insoles, I can pretty much do everything I want to do."* [P6, male, 50-60, toes/partial foot amputation, no prosthesis] | **Difficulties running*:**  *"Yes, I was at a prosthetic festival in Amsterdam recently, and there were people with, well, they at least still had a stump, you know, so they could wear a running prosthesis. It seems great to me, of course, but that’s not going to happen for me. So no, running, never again, that can never happen."* [P1, female, 50-60, bone, hindquarter amputation, prosthesis] |
| **Swimming/**  **Environmental factors/products and technology*/assistive products and technology for personal indoor and outdoor mobility and transportation** | **d4554/**  **e1201** |  | **Difficulties swimming*:**  *"Yes, unless I want to go swimming or something, then I need crutches again. But other than that, everything is with the prosthesis."* [P30, female, 30-40, bone, above knee amputation, prosthesis] |  |
| **Going up and down the stairs**  **Environmental factors/products and technology*/assistive products and technology for personal indoor and outdoor mobility and transportation** | **d451/**  **e1201** |  | **Difficulties walking stairs*:**  *"At home, I try to walk around as much as possible without support, except when I use the stair lift to go upstairs, since the bedroom is up there. And it’s going well, let me put it that way."* [P20, male, 80-90, STS, through knee amputation, prosthesis] |  |
| **Moving around using equipment/**  **Environmental factors/products and technology*/assistive products and technology for personal indoor and outdoor mobility and transportation** | **d465/**  **e1201** | **Use an e-bike*:** *"No, an electric bike. Though, of course, the brakes have been moved to one side, right?"* [P4, male, 70-80, STS, forequarter amputation, no prosthesis]  **Use a cargo bike*:**  ***“****I eventually got a cargo bike with three wheels. I rode that for a very long time."* [P7, female, 40-50, bone, disarticulation amputation, no prosthesis] | **Difficulties riding a bicycle*:**  *"The same applies here: one leg still works very well, but the other leg can only provide about 20 percent of the energy, I believe. That makes for a very awkward way of cycling. A lot of energy on one side, very little on the other. You really have to train yourself to... the weaker leg should actually take the lead. It needs to determine how much pressure you can apply. So the other leg should essentially do nothing, just pedal along without providing the power. The focus really needs to be on the weaker leg."* [P38, male, 60-70, bone, above knee amputation, prosthesis]  **Use a walker*:** *"I walk every day with a walker. (…) Yes, outside. Because luckily, I live outside. So I walk on the public road. And that goes fine. I walk quite a distance."* [P20, male, 80-90, STS, through knee amputation, prosthesis]  **Use a wheelchair*:**  *"And for outings, I use the wheelchair. To an amusement park or something like that, I sit in a wheelchair. And at a holiday park, I drive around with a wheelchair, often even in the cabin."* [P3, male, 30-40, bone, hindquarter amputation, no prosthesis]  **Use insoles in shoes*:**  *"Now it's more focused on figuring out how I can walk without pain. So, by shifting the pressure on the insoles constantly."* [P33, female, 40-50, STS, toes/partial foot, no prosthesis]  **Use special shoes***: *"I wear water shoes at home because they give me more grip."* [P3, male, 30-40, bone, hindquarter amputation, no prosthesis]  **Use of triple chair*:**  *"Now I sit on a... They call it a triple chair. A desk chair, but with that, you can relatively easily roll back and forth. So if, for example, I want something in the kitchen... I cook in the kitchen occasionally. Not very often, though, I'm not a big cook. But I do it from time to time. Then I actually just sit on that chair because it's much less tiring for me than constantly standing at the counter."* [P38, male, 60-70, bone, above knee amputation, prosthesis]  **Use an e-bike*:** *"With an e-bike, it becomes much easier again."* [P38, male, 60-70 above knee amputation, prosthesis]  **Use a stair lift*:**  *"And I have a stair lift at home. That’s another thing, because I said, 'I need that stair lift, because soon I’ll have a baby, and I won’t be able to carry the child upstairs."* [P3, male, 30-40, bone, hindquarter amputation, no prosthesis]  **Use handrails*:**  *"It’s just that my partner has installed handrails, because there are those sharp edges at the end of the banister that you can’t grip properly. So we have handrails on the upper side of the stairs to make it easier to hold on."* [P34, female, 30-40, bone, hindquarter amputation, no prosthesis] | **Use a wheelchair:** *"I do have a wheelchair, indeed."* [P1, female, 50-60, bone, hindquarter amputation, prosthesis]  **Use prosthesis for balance on hand bike*:**  *"I always put on my prosthesis when I hand bike, but purely for balance, you know. That leg weighs something too, so I don't have all the weight on the left side of the chair, but also on the right side.* [P1, female, 50-60, bone, hindquarter amputation, prosthesis]  **Use a hand bike:**  *"I have a hand bike."* [P1, female, 50-60, bone, hindquarter amputation, prosthesis] |
| **Driving** | **d475** | **Difficulties driving a car:**  *"But I also notice when I drive for a long time, that’s a recurring problem, I can feel it in my arm. (…) I do drive, but I don’t drive long distances."* [P7, female, 40-50, bone, disarticulation amputation, no prosthesis] |  |  |
| **Self-care (ICF category)** | [**ICF codes**](https://icd.who.int/browse/2025-01/icf/en#619527855) | **Upper extremity amputation**  **Theme** *quote* [study number, gender, age range, sarcoma type, amputation level, (no) prosthesis] | **Lower extremity amputation**  **Theme** *quote* [study number, gender, age range, sarcoma type, amputation level, (no) prosthesis] | **Pelvic amputation**  **Theme** *quote* [study number, gender, age range, sarcoma type, amputation level, (no) prosthesis] |
| **Dressing/Putting on clothes &**  **Other specified washing oneself/**  **Environmental factors/Support and relationship**/Immediate family**  **Environmental factors/products and technology*/assistive products and technology for personal indoor and outdoor mobility and transportation** | **d5400/**  **d5108/**  **e310/**  **e1201** | **Difficulties closing buttons:**  *"The only thing is, with buttons it's a bit more difficult. So, for example, if you have jeans with a button, that's harder than, for instance, a zipper."* [P4, male, 70-80. STS, forequarter amputation, no prosthesis]  **Difficulties putting on a bra:**  *"First of all, with getting dressed, I can't put on my bra."* [P2, female, 60-70, STS, disarticulation amputation, no prosthesis] | **Difficulties with dressing and showering**:**  *"Getting dressed and showering, my wife always helps a little with that."* [P28, male, 60-70, bone, through knee amputation, prosthesis] | **Difficulties with dressing*:**  *"Yeah, it's fine. I always have a chair or a bed, I usually change on the edge of my bed, of course."* [P1, female, 50-60, bone, hindquarter amputation, prosthesis] |
| **Putting on footwear** | **d4502** | **Difficulties tying shoelaces:**  *"Of course, very simple things like tying your shoelaces, that’s just impossible."* [P4, male, 70-80, STS, forequarter amputation, no prosthesis] |  |  |
| **Other specified washing oneself/**  **Environmental factors/products and technology*/assistive products and technology for personal indoor and outdoor mobility and transportation** | **d5108/**  **e1201** |  | **Use a bath plank*:**  *"Well, I have the bath board. (…) Because I sit on the edge of the bath on the left side, my partner helped me rinse that wound. But now I don't need help with that anymore, but I still use the bath board."* [P34, female, 30-40, bone, hindquarter amputation, no prosthesis]  **Use handrails to shower*:**  "*And further, I still use a handle here and there in the house. In the shower, I have a grab bar, and in the toilet as well, because it makes it easier to stand up. But that's more for convenience, because I could probably manage without, but it's just safer this way."* [P37, female, 70-80, STS, above knee amputation prosthesis]  **Use a chair to shower*:**  *"I had asked for a chair in the shower."* [P27, male, 70-80, bone, below knee amputation, prosthesis]  **Use special prosthesis in shower*:**  *"I've had a shower prosthesis for fourteen days now, with an extra rubber foot underneath. (…) Well, I really wanted a shower prosthesis. (…) And when I go in the shower, I attach the shower prosthesis."* [P37, female, 70-80, STS, above knee amputation, prosthesis] | **Use handrails to shower*:** *"But I do have handrails, so to speak, that I can hold onto."* [P1, female, 50-60, bone, hindquarter, amputation prosthesis] |
| **Caring for hair** | **d5202** | **Difficulties styling my hair:**  *“Combing my hair goes fine, but if I want a ponytail or a bun or something, then that’s something I can’t do. Because you really need two hands for that.* [P7, female, 40-50, bone, disarticulation amputation, no prosthesis] |  |  |
| **Other specified toileting/**  **Environmental factors/products and technology*/assistive products and technology for personal indoor and outdoor mobility and transportation** | **d5308/**  **e1201** |  | **Using handrails for toilet*:** *"And further, I still use a handle here and there in the house. In the shower, I have a grab bar, and in the toilet as well, because it makes it easier to stand up. But that's more for convenience, because I could probably manage without, but it's just safer this way."* [P37, female, 70-80, bone, above knee amputation, prosthesis]  **Using urinal in night*:** *"Look, you don't know this. I have a urinal next to my bed, because I don't wear the prosthesis. So I have to urinate in a urinal."* [P36, male, 70-80, bone, above knee amputation, prosthesis] | **Using handrails for toilet*:** *"I can't sit normally on the toilet. I always have to hold on because the right side doesn't reach the seat."* [P1, female, 50-60, bone, hindquarter amputation, prosthesis]  **Removing prosthesis before toileting*:**  *"So when I go to the toilet, I always have to take it off. So, you know, it's not like you can go to the toilet with it on."* [P1, female, 50-60, bone, hindquarter amputation, prosthesis] |
| **Domestic life (ICF category)** | [**ICF codes**](https://icd.who.int/browse/2025-01/icf/en#619527855) | **Upper extremity amputation**  **Theme** *quote* [study number, gender, age range, sarcoma type, amputation level, (no) prosthesis] | **Lower extremity amputation**  **Theme** *quote* [study number, gender, age range, sarcoma type, amputation level, (no) prosthesis] | **Pelvic amputation**  **Theme** *quote* [study number, gender, age range, sarcoma type, amputation level, (no) prosthesis] |
| **Household tasks/**  **Environmental factors/Support and relationship**/immediate family**  **Environmental factors/products and technology*/assistive products and technology for personal indoor and outdoor mobility and transportation** | **d469/**  **e310/**  **e1201** | **Difficulties working in the garden**:**  *"Oh yes, so then I got some clippers, you know, just to quickly snip a flower. Just regular garden clippers. You know, but I couldn’t get them to open. But I also had this... We have a little spot where we have a sort of garden as well, and I was just happily working there with a normal pair of clippers. And I said, ‘Yeah, that branch has to go too. I’ll go grab the clippers and take care of it.’ So I grabbed the big clippers. Well, I was standing there trying to cut off that branch, and I thought, my god, how on earth am I supposed to do this? Well, those kinds of things, you know. ‘Yeah,’ my husband says, ‘I’ll do it then.’"* [P2, female, 60-70, STS, disarticulation amputation, no prosthesis]  **Difficulties ironing clothes**:**  *"I can’t manage ironing either."* [P2, female, 60-70, STS, disarticulation amputation, no prosthesis]  **Difficulties changing bed sheets**:** *"Making the bed as well, you know, with a heavy mattress. Yeah, I just can’t manage that, so I have help for that too."* [P2, female, 60-70, STS, disarticulation amputation, no prosthesis]  **Difficulties preparing and serving food and drinks**:**  *"And serving. Yeah, my husband usually takes it off the stove and brings it inside. He says, 'I’d rather do it myself than something happen.' But sometimes I do put a pot on the table myself. And if it’s a pot with a handle, that’s much easier. (…) Yeah, like a wok pan. I’ve also seen, I’ve found some pans that you normally use with two handles, but this one has a single handle."* [P2, female, 60-70, STS, disarticulation amputation, no prosthesis] | **Difficulties ironing**:**  *"But my partner has to grab everything too—the iron, the laundry, and all the other things. (…) So you can do part of the task, but really not everything."* [P34, female, 30-40, bone, hindquarter amputation no prosthesis]  **Difficulties working in the garden*:**  *"Well, this week I was working in the garden again for the first time. Three weeks ago, I had also tried it with the prosthesis, but I struggled a lot back then. (…) Yeah, so, well, it’s not as easy as it used to be, you understand. Just a bit of weeding and things like that, which I wanted to do. But, yeah, you do have the whole day, I always say."* [P28, male, 60-70, bone, through knee amputation, prosthesis]  **Difficulties preparing and serving food or drinks**:** *"I can possibly do that while sitting, and serving… well, serving... To give a very practical example, I don’t take a casserole dish out of the oven. I leave that to my wife because I’m less stable when standing. Then my wife pushes me aside and says, 'I’ll do it for you.'"* [P38, male, 60-70, bone, above knee amputation, prosthesis] | **Difficulties with vacuuming*:** *"I can vacuum, but for me, it’s a full workout, and it’s not that big of a space, really. But yeah, you know, you hop and jump around on one leg, and I get back pain, you know, so it’s a bit limited."* [P1 bone hindquarter amputation, prosthesis]  **Difficulties preparing and serving food and drinks**:**  *"Cooking is indeed difficult for me, and serving is something I definitely can’t do. I can’t even carry my own cup of coffee."* [P1, bone, hindquarter amputation, prosthesis] |
| **Assisting others with self-care/**  **Environmental factors/products and technology*/assistive products and technology for personal indoor and outdoor mobility and transportation** | **d6601/**  **e1201** | **Difficulties caring for (grand) children:**  *"We also, yeah, every few weeks, we go babysit our new grandkids. That’s the problem: how do I get a baby out of the crib? (…) Because you just want to cuddle the baby, but it’s just placed in your arms. You can’t pull it close to you... It just sinks down, you know. But that was really difficult, yeah."* [P2, female, 60-70, STS, disarticulation amputation, no prosthesis] | **Difficulties caring for (grand) children*:** *"Or I used to have the baby carrier on my belly and walked on crutches with her in the back pocket. In the nursery, we had set up a kind of barber chair that could go up and down, so I could change her and stuff. (…) Yeah, sitting down, yeah. So that all worked fine. By now, she’s already four. Before, I always took her up the stairlift with me and had her on my lap. I’d put an arm around her and take her upstairs."* [P3, male, 30-40, bone, hindquarter amputation, no prosthesis] |  |
| **Community, social and civic life (ICF category)** | [**ICF codes**](https://icd.who.int/browse/2025-01/icf/en#619527855) | **Upper extremity amputation**  **Theme** *quote* [study number, gender, age range, sarcoma type, amputation level, (no) prosthesis] | **Lower extremity amputation**  **Theme** *quote* [study number, gender, age range, sarcoma type, amputation level, (no) prosthesis] | **Pelvic amputation**  **Theme** *quote* [study number, gender, age range, sarcoma type, amputation level, (no) prosthesis] |
| **Sports**  **Environmental factors/products and technology*/assistive products and technology for personal indoor and outdoor mobility and transportation** | **d9201/**  **e1201** |  | **Difficulties kickboxing*:**  *"So extreme sports are definitely not recommended anymore. I used to box, but I don’t do that anymore. Yeah, I used to do kickboxing, but I can’t do that now. I used to train five, six days a week, I was super athletic. I think I haven’t exercised for about a year now. That’s partly because of COVID, but kickboxing was always really my thing. Boxing just isn’t as fun for me, and now I’m still figuring out what sport I should do. I also really enjoy dancing, but with Zumba and things like that, it’s just a bit different. I just have to do my own moves and do my own thing when things don’t work out."* [P30, female, 30-40, bone, above knee amputation, prosthesis]  **Difficulties mountain biking*:**  *"And I used to go mountain biking, but more like touring on the road, just around my own area. So now it’s mostly handbiking. Yeah, it’s become hand biking now."* [P3, 30-40, bone hindquarter amputation, no prosthesis] | **Difficulties with dancing*:** *"Yeah, you know, dancing, for example, I can’t do that anymore."* [P1, female, 50-60, bone, hindquarter amputation, prosthesis] |
